# RNA sensor MDA5 suppresses LINE-1 retrotransposition by regulating the promoter activity of LINE-1 5′-UTR

**DOI:** 10.1186/s13100-022-00268-0

**Published:** 2022-04-12

**Authors:** Jiaxiu Yan, Yifei Zhao, Juan Du, Yu Wang, Shaohua Wang, Qing Wang, Xu Zhao, Wei Xu, Ke Zhao

**Affiliations:** 1 Institute of Virology and AIDS Research, First Hospital of Jilin University, Changchun, Jilin China; 2 Department of Neonatology, First Hospital of Jilin University, Changchun, Jilin China; 3 Department of Clinical Laboratory, First Hospital of Jilin University, Changchun, Jilin China; 4 Center for Pathogen Biology and Infectious Diseases, First Hospital of Jilin University, Changchun, Jilin China; 5 Key Laboratory of Organ Regeneration & Transplantation of the Ministry of Education, First Hospital of Jilin University, Changchun, Jilin China; 6 Department of Respiratory Medicine, First Hospital of Jilin University, Changchun, Jilin China; 7 Department of Hepatology, First Hospital of Jilin University, Changchun, Jilin China

**Keywords:** LINE-1, MDA5, Retrotransposition, 5′-UTR, Promoter regulation

## Abstract

**Background:**

Type 1 long interspersed elements, or LINE-1, are the only retroelements that replicate autonomously in human cells. The retrotransposition process of LINE-1 can trigger the activation of the innate immune system and has been proposed to play a role in the development of several autoimmune diseases, including Aicardi-Goutières syndrome (AGS). In contrast, all known AGS-associated proteins, except MDA5, have been reported to affect LINE-1 activity. Thus, MDA5 is likely to also function as a LINE-1 suppressor.

**Results:**

MDA5 was found to potently suppress LINE-1 activity in a reporter-based LINE-1 retrotransposition assay. Although MDA5 is an endogenous RNA sensor able to activate the innate immune system, increased interferon (IFN) expression only contributed in part to MDA5-mediated LINE-1 suppression. Instead, MDA5 potently regulated the promoter activity of LINE-1 5′-UTR, as confirmed by transiently expressed myc-tagged MDA5 or knockdown of endogenous MDA5 expression. Consequently, MDA5 effectively reduced the generation of LINE-1 RNA and the subsequent expression of LINE-1 ORF1p and ORF2p. Interestingly, despite MDA5 being a multi-domain protein, the N-terminal 2CARD domain alone is sufficient to interact with LINE-1 5′-UTR and inhibit LINE-1 promoter activity.

**Conclusion:**

Our data reveal that MDA5 functions as a promoter regulator; it directly binds to the LINE-1 5′-UTR and suppresses its promoter activity. Consequently, MDA5 reduces LINE-1 RNA and protein levels, and ultimately inhibits LINE-1 retrotransposition. In contrast, MDA5-induced IFN expression only plays a mild role in MDA5-mediated LINE-1 suppression. In addition, the N-terminal 2CARD domain was found to be a functional region for MDA5 upon inhibition of LINE-1 replication. Thus, our data suggest that besides being an initiator of the innate immune system, MDA5 is also an effector against LINE-1 activity, potentially forming a feedback loop by suppressing LINE-1-induced innate immune activation.

**Supplementary Information:**

The online version contains supplementary material available at 10.1186/s13100-022-00268-0.

## Background

RNA sensing plays an important role in innate immune activation inside human cells. It senses the presence of non-self RNA (viral RNA, for instance) and activates downstream pathways, leading to the expression of interferons (IFNs) [36]. Subsequently, IFN triggers the expression of interferon-stimulated genes (ISGs), many products of which function as suppressors against exogenous pathogens [23, 39]. One of the well-studied RNA sensors is melanoma differentiation-associated protein 5 (MDA5). MDA5 belongs to the RIG-I-like receptor (RLR) family and generally detects viral RNA. Once bound to a viral RNA, MDA5 activates the downstream mitochondrial antiviral signalling protein (MAVS) and ultimately triggers the activation of the innate immune system [8]. Therefore, it would be easy to imagine that the malfunction of MDA5 can trigger innate immune dysregulation. Mutations in MDA5 have consistently been associated with an autoimmune disease termed Aicardi-Goutières syndrome (AGS) [30, 35].

In addition to MDA5, there are six other proteins that have been linked with AGS [7]. Notably, among these proteins, TREX1, SAMHD1, and ADAR1 were found to suppress the replication of the type 1 long interspersed element (LINE-1 or L1) [24, 31, 42, 51]. However, whether the heterotrimer RNaseH2 formed by the other three AGS-associated proteins inhibits or supports LINE-1 replication remains a topic of controversy [4, 6]. In human cells, LINE-1 is the only active retrotransposon that can replicate autonomously [5]. A full-length LINE-1 element is 6-kb in length, containing two sense open reading frames (*ORF1* and *ORF2*) that are flanked by the 5′-untranslated region (5′-UTR) functioning as the promoter and the 3′-UTR containing the poly A signal [11, 15]. Both coded proteins (ORF1p and ORF2p, respectively) interact with LINE-1 RNA to trigger the assembly of other proteins to form the LINE-1 ribonucleoprotein particle (RNP), which is a fundamental unit for LINE-1 retrotransposition [21, 46]. In a recent study, we found that LINE-1 RNP also functions as an endogenous trigger to activate the innate immune system through RNA-sensing pathways [52], providing direct evidence of the relationship between retrotransposons, innate immune activation, and IFN-based autoimmune diseases.

Interestingly, one of the RNA sensors involved in the above pathways is MDA5 [52]. Another intriguing fact is that MDA5 is not only an initiator of increased IFN production, but also an effector of IFN activation (i.e., IFN promotes MDA5 expression) [18]. This most likely indicates that, in addition to sensing RNA, MDA5 may possess certain function(s) post-innate immune activation. Given the complex relationship between MDA5 and AGS, LINE-1 and innate immune activation, and IFN and MDA5, we hypothesized that MDA5 may function as a potent LINE-1 suppressor.

## Results

### MDA5 potently suppresses LINE-1 retrotransposition in cultured cells

**Fig. 1:**
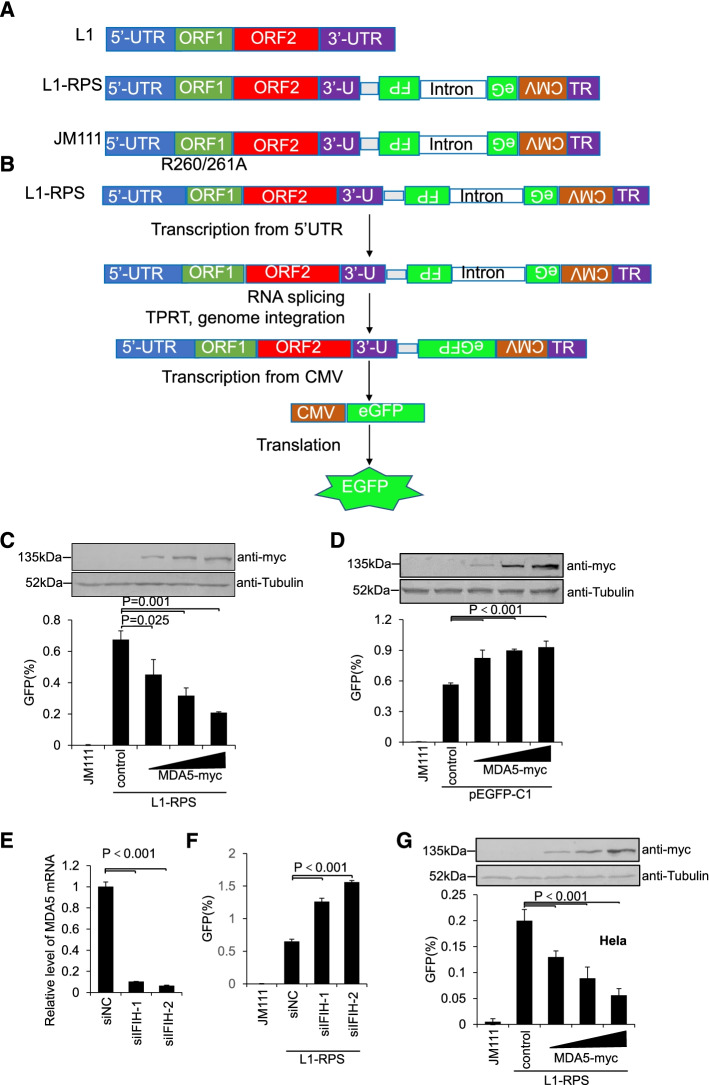
MDA5 suppresses LINE-1 activity in an EGFP-based LINE-1 retrotransposition assay. **A** LINE-1 expressing cassettes used in this study. In the retrotransposition-competent plasmid L1-RPS, an anti-sense CMV-EGFP cassette is inserted into the 3′-UTR of LINE-1, whereas the *EGFP* gene is interrupted by a sense Group I intron. JM111 is based on L1-RPS but contains an R260A/R261A mutation on ORF1p, and is therefore incompetent in retrotransposition. **B** Schematic representation of the mechanism of the EGFP-based LINE-1 assay. To produce an EGFP signal after transfection, L1-RPS needs to be transcribed through the LINE-1 5′-UTR promoter to generate the RNA, spliced to remove the intron, and then reverse-transcribed and integrated into the genome to finish the replication. Only then can the EGFP signal be generated through the expression of the integrated CMV-EGFP cassette. Any direct transcription of the CMV-EGFP cassette from the plasmid will not produce EGFP because of the un-spliced intron. **C** Flow cytometry results showing that MDA5-myc potently suppresses the retrotransposition activity of L1-RPS in HEK293T cells. HEK293T cells seeded on a 24-well plate were co-transfected with 1 μg of L1-RPS and control vector VR1012 (225 ng) or MDA5-myc-expressing vector (25 ng, 75 ng, and 225 ng). Cells were collected at 96 h post-transfection to detect EGFP-positive cells through flow cytometry. The Western blotting results above indicate the MDA5-myc protein levels in transfected cells. **D** Flow cytometry results showing that MDA5-myc does not suppress EGFP expression driven by the CMV promoter. HEK293T cells seeded on a 24-well plate were co-transfected with 45 ng of pEGFP-C1 and 225 ng of control vector VR1012 or 25, 75, or 225 ng of MDA5-myc-expressing vector. Cells were collected at 96 h post-transfection to detect EGFP-positive cells through flow cytometry. Western blotting results above indicate the MDA5-myc protein levels in transfected cells. **E** qRT-PCR results showing efficacy of *IFIH1*-specific siRNAs in HEK293T cells. HEK293T cells seeded on a 24-well plate were transfected with control 100 nM (final concentration) non-targeting control siRNA (siNC) or *IFIH1*-specific siRNAs (siIFIH1–1 and siIFIH1–2). Total RNA was extracted for each sample at 72 h post-transfection, and levels of endogenous *IFIH1* mRNA (encoding MDA5) were determined. **F** Flow cytometry results showing that LINE-1 activity is potently increased in HEK293T cells treated with IFIH1-specific siRNAs. HEK293T cells seeded on a 24-well plate were first treated with 100 nM siNC, siIFIH1–1 or siIFIH1–2, and then transfected with 1 μg of L1-RPS after 24 h. Cells were collected at 96 h post-transfection to detect EGFP-positive cells through flow cytometry. **G** Flow cytometry results showing that MDA5-myc potently suppresses the retrotransposition activity of L1-RPS in HeLa cells. HeLa cells seeded on a 24-well plate were co-transfected with 1 μg of L1-RPS and control vector VR1012 (225 ng) or MDA5-myc-expressing vector (25 ng, 75 ng, and 225 ng). Cells were collected at 96 h post-transfection to detect EGFP-positive cells through flow cytometry. The Western blotting results above indicate the MDA5-myc protein levels in transfected cells

To determine whether MDA5 suppresses LINE-1 activity, a widely used EGFP-based LINE-1 retrotransposition assay was introduced [28, 33]. This assay involves the use of two plasmids (Fig. 1A). Ninety-nine PUR RPS EGFP (L1-RPS) contains a sense LINE-1 element, whose 3′-UTR is interrupted with an antisense EGFP expression cassette, whereas the EGFP signal can only be detected as a post-retrotransposition event (see Methods for details) (Fig. 1B). Ninety-nine PUR JM111 EGFP (JM111) was similar to L1-RPS but contained two missense mutations on ORF1p, thus becoming retrotransposition-incompetent (Fig. 1A). A MDA5-myc plasmid was also constructed for the assay, which expresses wild-type MDA5 protein with a myc tag at its C-terminus (see Methods for detail). With the help of this LINE-1 retrotransposition assay, we found that exogenous MDA5-myc potently suppressed the replication of L1-RPS in HEK293T cells (Fig. 1C and Fig. S1A). Additional tests indicated that MDA5-myc did not reduce but slightly enhanced EGFP expression driven by the CMV promoter (Fig. 1D), suggesting that MDA5-myc indeed targeted LINE-1 for inhibition. To further confirm that endogenous MDA5 also functioned as a LINE-1 suppressor, we introduced two siRNAs specifically targeting *IFIH1* mRNA (which encodes the MDA5 protein) into the above LINE-1 retrotransposition assay. Both siRNA efficiently reduced endogenous *IFIH1* mRNA levels (Fig. 1E), while in the same cells the activity of L1-RPS retrotransposition was significantly increased (Fig. 1F), indicating that endogenous MDA5 indeed regulates LINE-1 replication. To rule out the possibility that MDA5-mediated LINE-1 suppression could only be specifically observed in HEK293T cells, LINE-1 retrotransposition was also performed in HeLa cells. Although LINE-1 replication rate was relatively lower, in part due to poor transfection efficiency and low retrotransposition competence of these HeLa cells [43], exogenous MDA5-myc protein remained effective in reducing LINE-1 activity in a dose-dependent manner (Fig. 1G and Fig. S1B). Thus, it appeared that MDA5 is a potent LINE-1 suppressor.

### IFN elevation, though unessential, contributes to MDA5-mediated LINE-1 suppression

**Fig. 2:**
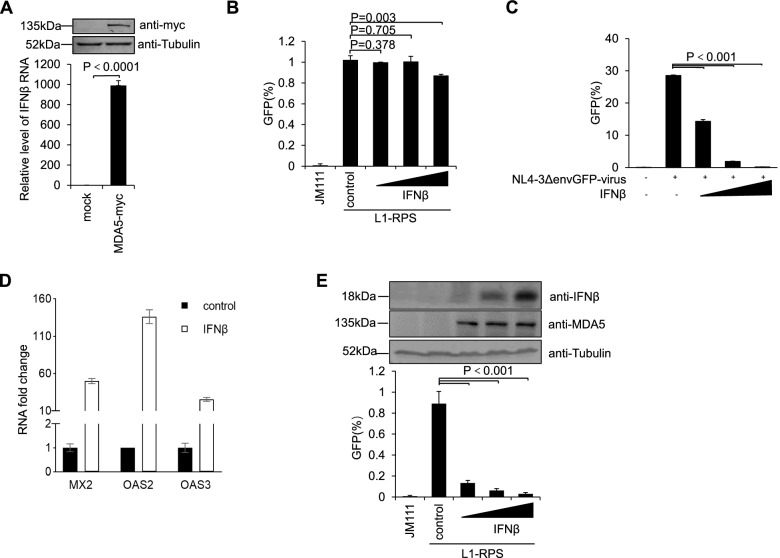
Intracellular instead of intercellular IFNβ induces suppressive effects against LINE-1 retrotransposition in HEK293T cells. **A** qRT-PCR data showing that exogenous MDA5 expression elevates IFNβ expression in HEK293T cells. HEK293T cells were transfected with 45 ng of MDA5-expressing vector and subjected to qRT-PCR at 48 h post-transfection. **B** Flow cytometry data indicating that the addition of IFNβ in culture medium does not induce LINE-1 suppression in HEK293T cells. HEK293T cells pre-treated with commercial IFNβ protein (2.5, 25, or 250 U/ml, final concentration) were transfected with 1 μg of L1-RPS. At 96 h post-transfection, the cells were collected to detect EGFP-positive cells through flow cytometry. **C** Flow cytometry data showing that IFNβ effectively induces HIV suppression in THP-1 cells. THP-1 cells pre-treated with commercial IFNβ protein (2.5, 25, or 250 U/ml, final concentration) were infected with VSVg-coated NL4–3 *Δenv* EGFP pseudovirus, and subjected to flow cytometry at 48 h post-infection to detect EGFP-positive cells. **D** qRT-PCR results showing that the expression of ISGs can be triggered by intracellular IFNβ. HEK293T cells were transfected with 135 ng of control vector VR1012 or 15, 45, or 135 ng of IFNβ-expressing vector, and subjected to qRT-PCR to detect endogenous levels of *MX2*, *OAS2*, and *OAS3* mRNA. Endogenous *ACTB* mRNA levels were used to equilibrate the results, but are not shown. **E** Flow cytometry data suggesting that intracellular IFNβ induces suppressive effects against LINE-1 retrotransposition in HEK293T cells. HEK293T cells seeded on a 24-well plate were co-transfected with 1 μg of L1-RPS and 225 ng of control vector VR1012 or 25, 75, or 225 ng of IFNβ-expressing vector. Cells were collected at 96 h post-transfection to detect EGFP-positive cells through flow cytometry. Western blotting results above indicate the IFNβ and MDA5 protein levels in transfected cells

MDA5 is the initiator of the RNA sensing pathway that triggers the activation of the innate immune system [18, 19]. The resulting elevation of IFN may induce the expression of ISGs, some of which have been identified as LINE-1 suppressors [12, 25, 51]. To determine whether MDA5 reduced LINE-1 retrotransposition through IFN elevation, we first checked and discovered that exogenous MDA5-myc expression indeed increased endogenous IFNβ production (Fig. 2A). IFNβ is a secreted protein that is believed to act normally by binding the receptor on the surface of a cell [45]. However, the treatment of HEK293T cells with IFNβ did not affect the retrotransposition potency of L1-RPS (Fig. 2B), whereas similar levels of IFNβ significantly reduced HIV infection in THP-1 cells (Fig. 2C). This was consistent with previous observations, wherein the treatment of HEK293T cells with IFNβ did not induce an antiviral effect [3].

Surprisingly, expressing exogenous IFNβ inside HEK293T cells significantly increased the expression levels of ISGs like MX2, OAS2, and OAS3, as indicated in the changes of endogenous mRNA levels with quantitative real-time polymerase chain reaction (qRT-PCR) (Fig. 2D). Notably, among these gene products, MX2 has been identified as a potent LINE-1 suppressor [13]. As a result, the expression of exogenous IFNβ in HEK293T cells effectively reduced the retrotransposition activity of L1-RPS (Fig. 2E). These data suggest that increasing endogenous IFNβ protein levels in HEK293T cells could inhibit LINE-1 replication, most likely by inducing ISG expression through intracellular pathway(s). Notably, endogenous levels of MDA5 protein were also increased in these cells (Fig. 2E), which was reasonable because MDA5 is also an ISG protein [40]; it also suggested that MDA5 plays a role in IFNβ-induced LINE-1 suppression.

**Fig. 3:**
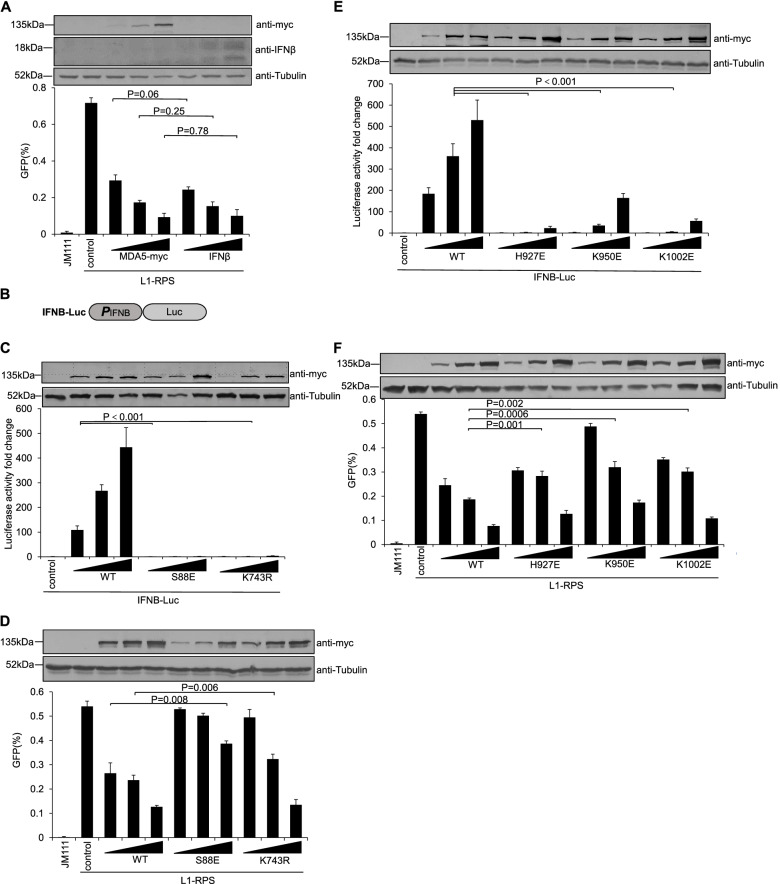
IFNβ elevation contributes mildly to MDA5-mediated LINE-1 suppression. **A** Flow cytometry data suggesting that exogenous MDA5-myc suppresses L1-RPS retrotransposition without significantly enhance endogenous IFNβ expression. HEK293T cells seeded on a 24-well plate were co-transfected with 1 μg of L1-RPS and 225 ng of control vector VR1012 or 25, 75, or 225 ng of MDA5-myc- or IFNβ-expressing vector. Cells were collected at 96 h post-transfection to detect EGFP-positive cells through flow cytometry. Western blotting results above indicate IFNβ and MDA5 protein levels in transfected cells. **B** Schematic representation of the IFNB-Luc expressing cassette (in the backbone vector pGL3-Basic), where the expression of firefly luciferase is driven by the *IFNB* promoter. **C** and **E** Luciferase activity data indicating the potency of wild-type MDA5-myc or its mutants in IFNβ elevation. HEK293T cells seeded in a 24-well plate were co-transfected with 100 ng of IFNB-Luc and 45 ng of control vector VR1012 or one of MDA5-myc-expressing vectors (5, 15, or 45 ng). Luciferase activity was tested at 48 h post-transfection. The western blotting results above indicate the MDA5 protein levels in transfected cells. **D** and **F** Flow cytometry results showing the efficacy of MDA5 mutants in LINE-1 suppression. HEK293T cells seeded on a 24-well plate were co-transfected with 1 μg of L1-RPS and control vector VR1012 (225 ng) or one of MDA5-myc-expressing vectors (25, 75, 225 ng). Cells were collected at 96 h post-transfection to detect EGFP-positive cells through flow cytometry. The western blotting results above indicate the MDA5-myc protein levels in transfected cells

To evaluate the contribution of IFN elevation in MDA5-mediated LINE-1 suppression, exogenous MDA5-myc and IFNβ were tested side-by-side for their potency in LINE-1 suppression. As shown in Fig. 3A, under similar levels of LINE-1 inhibition, MDA5-myc scarcely increased endogenous IFNβ expression when exogenous IFNβ was readily detected (Fig. 3A), which suggested that IFN activation might not be the key mechanism in MDA5-mediated LINE-1 suppression. To further confirm such a hypothesis, we introduced mutations on MDA5-myc to compromise the latter’s potency in IFN activation, which was tested in a promoter activity assay based on the expression of firefly luciferase (Fig. 3B). As a result, wild-type MDA5-myc effectively increased levels of luciferase expression driven by an *IFNB* promoter (IFNB-Luc) (Fig. 3C). Notably, this was not an off-target effect, because in similar tests MDA5 did not increase luciferase expression driven by the endogenous MCSFR promoter (MCSFR-Luc) or a promoter from the respiratory syncytial virus (RSV-Luc) (Fig.S2). On the other hand, MDA5 mutants, such as S88E and K743R that have been previously reported with reduced efficiency in innate immune activation [22, 47], were defective in activating *IFNB* promoter in HEK293T cells (Fig. 3C). However, although mildly ineffective, both S88E and K743R still suppressed LINE-1 retrotransposition (Fig. 3D). To further test this idea, more mutants were introduced in this study. RNA interaction is essential for the activation of MDA5 and the subsequent pathway to trigger IFN production, whereas amino acid residues such as H927, K950, and K1002 are important for MDA5’s efficacy for binding RNA [48]. Consistently, MDA5 mutants like H927E, K950E, or K1002E weakly activated the innate immune system (Fig. 3E). However, similar to S88E and K743R, these three mutants inhibited LINE-1 retrotransposition in HEK293T cells, with a slightly weakened potency compared to that of wild-type MDA5 (Fig. 3F). Therefore, although IFN elevation and the subsequent increased levels of ISG proteins contribute to MDA5-mediated LINE-1 suppression, a more effective mechanism should be applied.

### Levels of LINE-1 proteins are reduced in the presence of MDA5

**Fig. 4:**
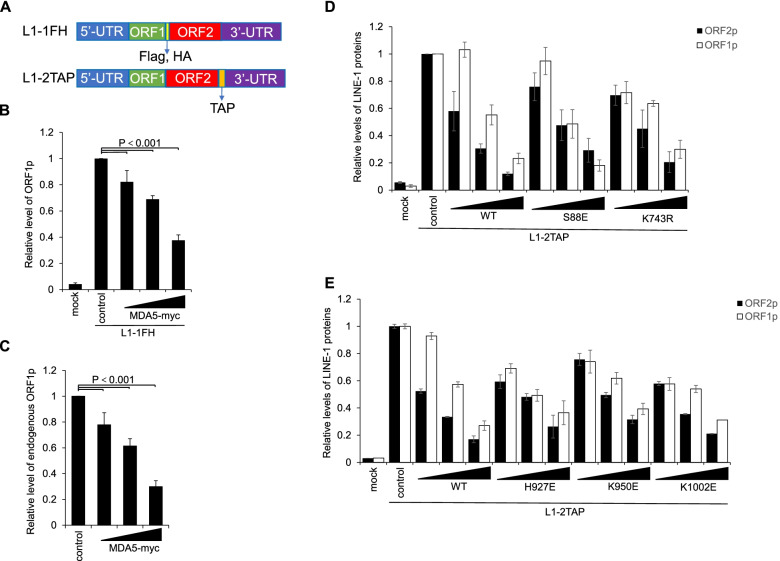
MDA5-myc reduces the expression levels of LINE-1 ORF1p and ORF2p. **A** Schematic representation of LINE-1 expression cassettes in L1–1FH and L1–2TAP, both of which were generated with pcDNA6/myc-His B as the backbone vector. L1–1FH expresses ORF1p with a Flag and HA tag at the C-terminus, and L1–2TAP expresses ORF2p with a TAP tag at the C-terminus. **B** Bar chart showing relative protein levels of Western blotting results that indicate MDA5-myc’s effect on the ORF1p expressed from L1–1FH. HEK293T cells seeded in a 24-well plate were co-transfected with 1 μg of L1–1FH and 225 ng of control vector VR1012 or 25, 75, or 225 ng of MDA5-myc-expressing vector, and subjected to western blotting at 48 h post-transfection. **C** Bar chart showing relative protein levels of Western blotting results that indicate MDA5-myc’s effect on endogenous ORF1p protein levels. HEK293T cells seeded in a 24-well plate were co-transfected with 1 μg of L1–1FH and 225 ng of control vector VR1012 or 25, 75, or 225 ng of MDA5-myc-expressing vector and subjected to western blotting at 48 h post-transfection. **D** and **E** Bar charts showing relative protein levels of Western blotting results that indicate the effects of MDA5-myc or its mutants on the expression levels of ORF1p and ORF2p-TAP expressed from L1–2TAP. HEK293T cells seeded in a 24-well plate were co-transfected with 1 μg of L1–2TAP and 225 ng of control vector VR1012 or 25, 75, or 225 ng of one of the vectors expressing wild type MDA5-myc or its mutants. Transfected cells were subjected to western blotting at 48 h post-transfection. All bar charts in this figure were generated based on relative levels of ORF1p and/or ORF2p of three independent experiments, and are shown as means ± SEM. Presentative western bloting results for each panel are shown in Fig. S3

Next, we investigated the mechanism underlying the MDA5-mediated suppression of LINE-1. It is worth noting, however, that despite MDA5 interacting with LINE-1 RNA [52], the above results with H927E, K950E, or K1002E mutants indicated that RNA binding may not contribute to MDA5’s potency in LINE-1 regulation. Therefore, we first tested whether MDA5 could affect the LINE-1 protein levels. L1–1FH was constructed based on the L1_RP_ sequence, expressing an ORF1p with an HA and a Flag tag at its C-terminus [12] (Fig. 4A). The co-transfection of L1–1FH and MDA5-expressing vector in HEK293T resulted in a reduction of ORF1p levels expressed from L1–1FH (Fig. 4B, and Fig.S3A) This was further confirmed by the observation that exogenous MDA5-myc expression led to the downregulation of endogenous ORF1p levels (Fig. 4C, and Fig. S3B).

We then generated another LINE-1 construct, L1–2TAP, into this study to further examine whether MDA5 could affect the expression/stability of ORF2p. Similar to L1–1FH, L1–2TAP was also constructed based on the L1_RP_, but expressing an ORF2p with a tandem affinity purification (TAP) tag at its C-terminus (Fig. 4A). Again, the expression of exogenous MDA5-myc protein reduced the levels of both untagged ORF1p and ORF2p-TAP expressed from L1–2TAP (Fig. 4D, and Fig. S3C).

To further validate whether the MDA5-mediated reduction of LINE-1 proteins was due to the MDA5-induced production of IFN, we also checked LINE-1 protein levels expressed from exogenous L1–2TAP under the presence of MDA5 mutants ineffective in innate immune activation. As shown in Fig. 4D and E (and Fig. S3C and D), MDA5 mutants including S88E, K743R, H927E, K950E and K1002E that were ineffective in activating the RNA sensing pathway or binding RNA remained potent in suppressing the expression of both ORF1p and ORF2p-TAP in HEK293T cells. These results indicated that MDA5 is potent in reducing the levels of LINE-1 ORF1p and ORF2p, to which MDA5-mediated innate immune activation does not contribute.

### MDA5 suppresses the promoter activity of LINE-1 5′-UTR

**Fig. 5:**
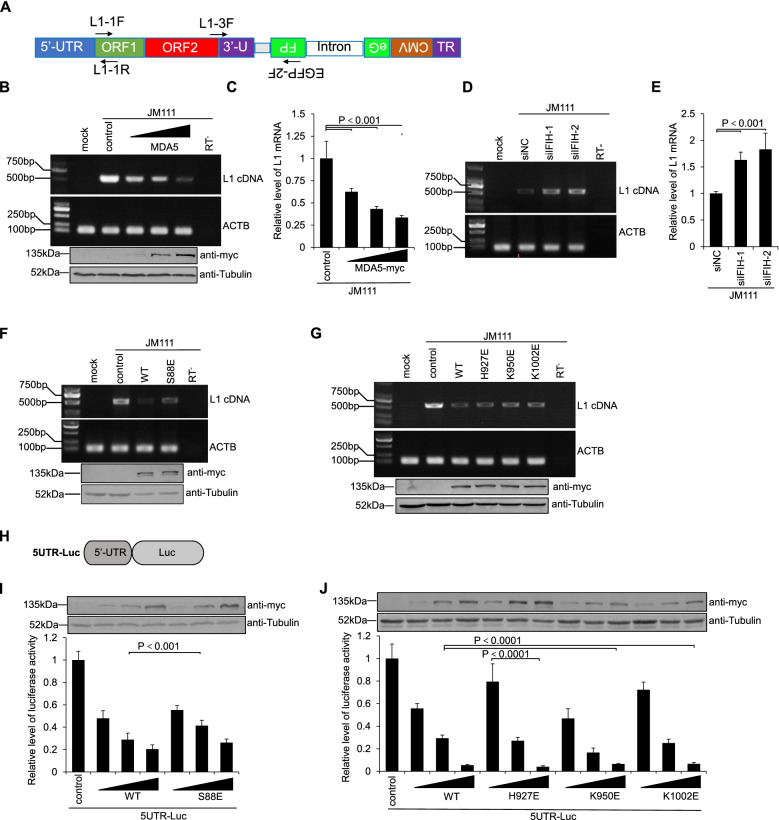
MDA5 suppresses the promoter activity of LINE-1 5′-UTR. **A** Schematic representation of the amplicon regions on JM111 in the PCR and qRT-PCR assay detecting LINE-1 RNA. Primers L1–3F and EGFP-2F target cDNA derived from JM111 RNA, and the amplified fragment represent full-length JM111 RNA, while L1–1F and L1–1R target cDNA derived from all LINE-1 mRNA (including endogenous LINE-1 mRNA and exogenous JM111 RNA). **B** Electrophoresis image showing that MDA5-myc reduces levels of LINE-1 RNA generated from JM111. HEK293T cells seeded in a 24-well plate were co-transfected with 1 μg of JM111 and 225 ng of control vector VR1012 or 25, 75, or 225 ng of MDA5-myc-expressing vector, and subjected to the PCR assay at 12 h post-transfection to detect the levels of LINE-1 RNA from JM111. The western blotting results show the MDA5-myc protein levels in the transfected cells. **C** qRT-PCR results based on samples from panel B showing that exogenous MDA5-myc reduces levels of total LINE-1 mRNA. **D** Electrophoresis image showing that knocking down endogenous MDA5 expression increases levels of LINE-1 RNA generated from JM111. HEK293T cells seeded in a 24-well plate were first transfected with 100 nM siNC, siIFIH1–1, or siIFIH1–2, and then transfected with 1 μg of JM111 after 24 h. Transfected cells were subjected to the PCR assay at 12 h post-transfection to detect the levels of LINE-1 RNA from JM111. **E** qRT-PCR results based on samples from panel D showing that knocking down endogenous MDA5 expression enhances levels of total LINE-1 mRNA. **F** and **G** Electrophoresis image showing the potency of MDA5-myc mutants in reducing the levels of LINE-1 RNA generated from JM111. HEK293T cells seeded in a 24-well plate were co-transfected with 1 μg of JM111 and control vector VR1012 (225 ng) or one of MDA5-myc-expressing vectors (225 ng) and subjected to the PCR assay at 12 h post-transfection to detect the levels of LINE-1 RNA from JM111. The western blotting results show the MDA5 protein levels in the transfected cells. **H** Schematic representation of the 5UTR-Luc cassette, which was generated with the backbone vector pGL3-Basic. **I** and **J** Luciferase activity data indicating the potency of wild type MDA5-myc or its mutants in LINE-1 5′-UTR regulation. HEK293T cells seeded in a 24-well plate were co-transfected with 200 ng of 5UTR-Luc and control vector VR1012 (225 ng) or one of MDA5-myc-expressing vectors (225 ng). Luciferase activity was tested at 48 h post-transfection. The western blotting results show the MDA5-myc protein levels in the transfected cells

It is worth noting that LINE-1 uses a single transcript to express both ORF1p and ORF2p proteins (Fig. 5A). Thus, a simultaneous reduction of both ORF1p and ORF2p expression most likely indicated a change in the levels of LINE-1 RNA. To test this hypothesis, L1-RPS and MDA5-myc expressing vector were co-transfected into HEK293T cells, and total RNA (containing L1-RPS transcripts) was extracted and reverse transcribed at 12 h post-transfection, a time point previously reported suitable for the determination of LINE-1 RNA levels [44]. As shown in Fig. 5B, the exogenous expression of wild-type MDA5-myc significantly reduced the levels of L1-RPS RNA (i.e., LINE-1 RNA transcribed from the L1-RPS plasmid), which was also confirmed by examining levels of total LINE-1 RNA (i.e., both endogenous LINE-1 RNA and exogenous L1-RPS RNA) with LINE-1-specific primers (Fig. 5C). On the contrary, shutting down endogenous MDA5 with *IFIH1*-specific siRNA increased the levels of L1-RPS RNA or total LINE-1 RNA (Fig. 5D and E). Interestingly, MDA5 mutant S88E ineffective in innate immune activation was only mildly inefficient in reducing L1-RPS RNA (Fig. 5F), and those MDA5 mutants incapable of binding RNA also maintained most if not all potency in reducing L1-RPS RNA (Fig. 5G). Notably, all the tested wild-type MDA5-myc and their mutants were effective in suppressing LINE-1 ORF1p and ORF2p expression (Fig. 4). Therefore, these data confirmed that MDA5 compromises the expression of ORF1p and ORF2p by reducing LINE-1 mRNA levels.

Intriguingly, the abovementioned data also suggested that RNA destabilisation may not be the reason for MDA5-mediated LINE-1 mRNA reduction, as MDA5 mutants that were unable to interact with RNA still showed efficiency in downregulating LINE-1 mRNA (Fig. 5G). As such, we focused on the LINE-1 5′-UTR, and tested whether MDA5-myc could affect the promoter activity of LINE-1 5′-UTR in a firefly luciferase reporter assay [24] (Fig. 5H). The results are shown in Fig. 5I and J. The luciferase activity detected in 5UTR-Luc-transfected HEK293T cells was lowered in the presence of MDA5-myc, indicating that MDA5 indeed compromised luciferase expression driven by the LINE-1 5′-UTR. Again, MDA5 mutants that were effective in LINE-1 suppression were also competent in 5′-UTR suppression (Fig. 5I and J), confirming that at least 5′-UTR suppression is major contributor to MDA5-mediated LINE-1 inhibition.

To further validate the idea that 5′-UTR regulation contributes to MDA5-mediated LINE-1 suppression, an idea, a new LINE-1 reporter plasmid ZY101 was reconstructed, which basically replaces the 5′-UTR in LINE-1 expressing cassette from L1-RPS with a CMV promoter (please refer to Method for more details) (Fig. S4A). Results from LINE-1 retrotransposition assay with ZY101 indicated that, although MDA5-myc did not affect CMV promoter activity, it only moderately inhibited the retrotransposition activity of ZY101 (Fig. S4B). This not only suggested that additional mechanism(s) might be involved in MDA5-mediated LINE-1 suppression, but also confirmed that 5′-UTR regulation is indeed a mechanism for MDA5 to inhibit LINE-1 replication.

### The 2CARD region is important for MDA5-mediated 5′-UTR regulation

**Fig. 6:**
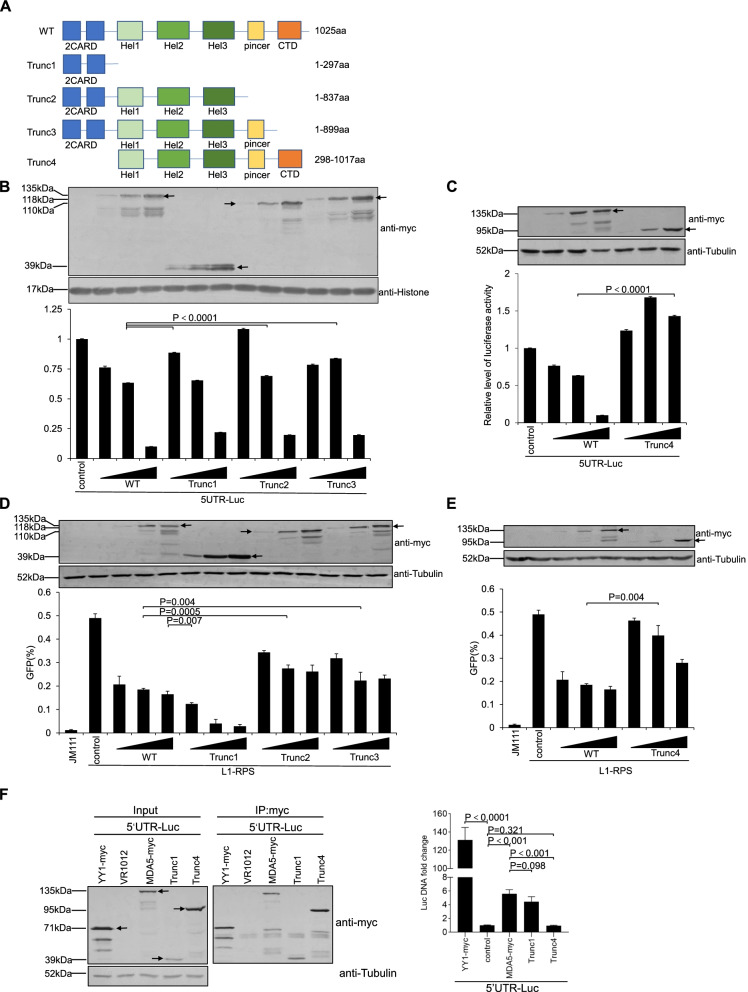
The 2CARD region is critical for MDA5-mediated LINE-1 suppression. **A** Schematic representation showing the deletion mutants of MDA5. **B** and **C** Luciferase activity data indicating the potency of MDA5-myc mutants in LINE-1 5′-UTR regulation. HEK293T cells seeded in a 24-well plate were co-transfected with 200 ng of 5’UTR-Luc and control vector VR1012 (225 ng) or one of MDA5-myc-expressing vectors (25, 75, or 225 ng). Luciferase activity was tested at 48 h post-transfection. The western blotting results show the MDA5-myc protein levels in the transfected cells. **D** and **E** Flow cytometry results showing the efficacy of MDA5-myc mutants in LINE-1 suppression. HEK293T cells seeded on a 24-well plate were co-transfected with 1 μg of L1-RPS and control vector VR1012 (225 ng) or one of MDA5-myc-expressing vectors (25, 75, or 225 ng), and were collected at 96 h post-transfection to detect EGFP-positive cells through flow cytometry. The western blotting results indicate the MDA5-myc protein levels in the transfected cells. **F** Co-IP experiment results indicating that MDA5 can interact with 5UTR-Luc plasmid DNA. HEK293T cells seeded on 6-well plate were co-transfected with 5UTR-Luc (800 ng) and another vector expressing YY1-myc (1200 ng), MDA5-myc (1200 ng), Trunc1 (1200 ng), Trunc4 (1200 ng), or the control vector VR1012 (1200 ng). Co-IP was performed at 48 h post transfection. DNA was extracted from eluted samples and subjected to qRT-PCR with primers targeting the luciferase gene. The multi-band phenomena detected for MDA5 in panels B-F are similar to previous reported observations [37]. Arrows are used to indicate bands of interested proteins with predicted sizes for YY1-myc, wild type MDA5-myc, and its mutants in these panels

We then continued our search for the region(s) of MDA5 that might be involved in 5′-UTR suppression. The human MDA5 protein is a 1025-amino acid-long protein with multiple regions (Fig. 6A). To identify the region that is essential for MDA5-mediated 5′-UTR regulation, continuous deletions were made from the N- or C-terminus of MDA5-myc (Fig. 6A), and the resulting mutants were tested for their efficacy to inhibit 5′-UTR. Interestingly, C-terminal deletions that left the 2CARD (tandem caspase activation and recruitment domain) region (Trunc1, 2, and 3) untouched only weakly affected MDA5’s potency in 5′-UTR suppression (Fig. 6B), whereas deleting 2CARD alone (Trunc4) completely abolished MDA5-myc’s efficiency to regulate LINE-1 5′-UTR (Fig. 6C). Consistently, the 2CARD region alone was sufficient to reduce the retrotransposition activity of L1-RPS (Fig. 6D). It was noteworthy that a similar 2CARD region from another RNA sensor RIG-I can induce IFN production [29], and similar phenomenon was also detected with the same region from MDA5 (Fig. S5A). However this was not the reason for this region to suppress LINE-1 replication, because introducing S88E mutation to MDA5’s 2CARD completely abolished 2CARD’s ability to activate the *IFNB* promoter but barely reduced its efficacy in regulating LINE-1 (Fig. S5). On the other hand, losing 2CARD significantly compromised the capability of MDA5 to suppress LINE-1 replication (Fig. 6E). Notably, Trunc4 still possessed low level potency in regulating LINE-1, which correlated with our previous observation that MDA5 could suppress LINE-1 activity through at least one mechanism that is independent from MDA5-induced IFN production or 5′-UTR inhibition (Fig. S4). Additional tests indicated that, both MDA5-myc and Trunc1 that were effective in regulating LINE-1 5′-UTR were capable of binding the 5UTR-Luc plasmid, which could not be observed with Trunc4 which did not inhibit 5′-UTR (Fig. 6F), suggesting MDA5 most likely regulates LINE-1 promoter activity through direct interaction with LINE-1 5′-UTR. In combination, these data suggest that, the 2CARD region is critical for MDA5 to suppress the promoter activity of LINE-1 5′-UTR and is important for MDA5-mediated LINE-1 suppression.

## Discussion

Recent studies have increasingly indicated that the activity of endogenous retroelements, such as LINE-1, is associated with autoimmune diseases, such as AGS [42, 52], Sjögren’s syndrome [27], and systemic lupus erythematosus [17], most likely by activating the innate immune system [1, 14, 26, 34, 49]. Consistently, many AGS-associated proteins, such as TREX1, SAMHD1, and ADAR1, are potent LINE-1 suppressors [24, 31, 42, 51]. In this study, we identified another AGS-associated protein, MDA5, which is a LINE-1 regulator. Indeed, both exogenous MDA5-myc and endogenous MDA5 effectively reduced the retrotransposition activity of L1-RPS in different cells. Further investigations revealed that MDA5 decreased the protein levels of both ORF1p and ORF2p, as the presence of MDA5 lowered the levels of LINE-1 RNA. Interestingly, although MDA5 was found to be capable of binding LINE-1 RNA to induce the activation of the innate immune system [52], RNA binding capacity was not the reason for the MDA5-induced reduction in LINE-1 RNA. Instead, MDA5 suppressed the promoter activity of LINE-1 5′-UTR with its N-terminal 2CARD region, which ultimately resulted in the inhibition of LINE-1 retrotransposition.

As an RNA sensor, MDA5 triggers the expression of endogenous IFN. Surprisingly, despite the widely accepted idea that IFNs are secreted proteins that normally function by binding IFN receptors on the cell surface [45], treating HEK293T cells with extracellular IFNβ only weakly affected LINE-1 replication. Instead, expressing IFNβ inside HEK293T cells significantly suppressed LINE-1 replication, suggesting the existence of intracellular pathway(s) of IFN-inducing ISG expression. Indeed, further examination confirmed that intracellular IFNβ could increase the expression of some ISGs, whereas some of these gene products are potent LINE-1 suppressors. However, it appeared that activating IFN production only had a partial contribution to MDA5-mediated LINE-1 suppression, as MDA5 mutants that failed in trigging innate immune activation maintained most effect on LINE-1 activity. This, in contrast, correlated with the fact that all these mutants were still fully functional in reducing the promoter activity of LINE-1 5′-UTR, as well as the subsequent expression of LINE-1 proteins, confirming that MDA5 represses LINE-1 retrotransposition mostly through the disruption of the efficacy of LINE-1 5′-UTR.

The abovementioned data suggest that besides sensing RNA and triggering IFN production through the RNA sensing pathway, MDA5 may also have other role(s). In other words, in addition to being an initiator, MDA5 may also be an effector or even a regulator of the innate immune activation, which is consistent with the fact that MDA5 is one of the ISGs [40]. Indeed, in this study, IFNβ-mediated LINE-1 suppression is accompanied with elevation of endogenous MDA5 proteins. On the other hand, MDA5 potently suppressed the promoter activity of LINE-1 5′-UTR through a mechanism independent of IFN elevation. Considering that endogenous retroelements are believed to be fossils of ancient retroviruses [11], it is possible that, after being upregulated post-innate immune activation, MDA5 could function as a restriction factor to suppress gene expression from exogenous pathogens. It is also likely that elevated MDA5 may affect the levels of host RNA and/or proteins, to either generate additional anti-pathogen defence or regulate the levels of innate immune activation. In fact, MDA5 was first discovered through its ability to induce apoptosis [18, 20], which leads to the elimination of infected cells [32]. In contrast, it was previously reported (and confirmed in our study) that expressing exogenous MDA5 alone can increase endogenous IFN levels [2], constituting a feed-forward loop. Our study also suggested another pathway through which, due to lower levels of LINE-1 RNA and proteins, as well as subsequent LINE-1 retrotransposition, MDA5 downregulated LINE-1-induced innate immune activation, forming a feedback control of the innate immune system, at least to some degree.

However, surprisingly, the 2CARD region is important for MDA5-mediated LINE-1 suppression, because the oligomerization of the same region is also essential for the activation of MAVS, as well as the subsequent RNA-sensing pathway, leading to the production of IFN [48]. Indeed, 2CARD by itself is sufficient to reduce the promoter activity of the 5′-UTR, as well as the expression levels of both LINE-1 proteins. Interestingly, it appeared that 2CARD may activate the RNA sensing pathway and suppress LINE-1 activity through different interfaces. The amino acid residue S88 in the first CARD region functions as a switch for the MDA5-mediated RNA sensing pathway; when S88 is phosphorylated by phosphatase PP1 or mutated to amino acids, such as aspartic acid (D) and glutamic acid (E), which mimic phosphorylation, MDA5 no longer activates the innate immune system [47]. Consistently, we also confirmed that the S88E mutation significantly compromises MDA5’s ability to activate the IFNB promoter. However, S88E did not affect MDA5-mediated LINE-1 suppression. Indeed, MDA5 S88E is fully capable of reducing the promoter activity of LINE-1 5′-UTR and subsequently decreasing levels of LINE-1 ORF1p and ORF2p. Therefore, although 2CARD is essential for MDA5 to promote IFN interaction and to suppress LINE-1 5′-UTR, different critical residues are used in this region to activate the innate immune system or repress LINE-1 activity.

Intriguingly, although we focused on MDA5′-mediated suppression of LINE-1 5′-UTR, it appeared that MDA5 can suppress LINE-1 retrotransposition through multiple mechanisms. For instance, despite the less efficacy, it seemed that MDA5 can also regulate LINE-1 replication through IFN promotion. Indeed, comparing to those mutants (such as S88E) that cannot trigger IFN activation, wild type MDA5-myc is more effective in 5′-UTR regulation and LINE-1 suppression. Another example is Trunc4, which loses the 2CARD region essential for innate immune activation and has no effect on 5′-UTR repression. However, Trunc4 still possesses a weakened potency in suppressing LINE-1 retrotransposition. This might indicate the importance not only of LINE-1 regulation for human cells but also of MDA5’s role as a LINE-1 suppressor; details of both are waiting to be further explored.

## Conclusions

MDA5 was found to be a potent LINE-1 suppressor. As an initiator of the RNA sensing pathway, MDA5 can trigger the production of IFN and subsequent ISGs, some of which function as LINE-1 inhibitors. In contrast, as an effector of innate immune activation, MDA5 can also suppress the promoter activity of LINE-1 5′-UTR in an IFN-independent mechanism, leading to the reduction of LINE-1 RNA and proteins, as well as the inhibition of LINE-1 retrotransposition. Although the same 2CARD region is critical for MDA5-mediated innate immune activation and promoter suppression, different interfaces or at least different amino acid residues are used in both functions. Thus, by uncovering the mechanism of MDA5-mediated LINE-1 suppression, we have revealed that promoter suppression is a novel function of MDA5, the study of which will extend our understanding of the biological functions of MDA5.

## Materials and methods

### Cells and plasmids

Human embryo kidney 293 T (HEK293T) cells were cultured with DMEM (Thermo Fisher Scientific, USA) supplemented with 10% fetal bovine serum (Thermo Fisher Scientific, USA), in an incubator containing 5% CO_2_ under 37 °C.

The *IFIH1* (encoding MDA5) gene was retrieved from HEK293T cells through reverse transcription and polymerase chain reaction (PCR), and inserted into VR1012 (containing a CMV promoter/enhancer, intronA, multiple cloning sites, and BGH polyA signal sequence) [16] with the help of SalI and BamHI endonucleases. The generated plasmid expresses wild-type MDA5 with a myc-tag at the protein’s C-terminus (MDA-myc). Point mutations of MDA5-myc were achieved using standard site-directed mutagenesis techniques, and truncations were achieved through PCR based on the MDA5-myc plasmid. All mutants constructs were sequence confirmed.

The *YY1* gene was retrieved from HEK293T cells through reverse transcription and polymerase chain reaction (PCR), and inserted into VR1012. The generated plasmid expresses wild-type YY1 with a myc-tag at the protein’s C-terminus (YY1-myc).

The retrotransposition-competent vector 99 PUR RPS EGFP (L1-RPS) [33], the retrotransposition incompetent 99 PUR JM111 EGFP (JM111) [33], pc-L1–1FH (L1–1FH) [12], pGL3–5′-UTR-luciferase (5UTR-Luc) [24], and pGL3-IFNB-luciferase (IFNB-Luc) [41] have been described previously. pGL3-MCSFR-Luciferase (MCSFR-Luc) and pGL3-RSV-Luciferase (RSV-Luc) were kind gifts from Dr. Xiao-Fang Yu, where the *MCSFR* promoter and the RSV promoter are inserted into pGL3-Basic (Promega, Madison, WI), respectively.

pEGFP-C1 was purchased from Clontech.

The pc-L1–2TAP (L1–2TAP) was constructed through DNA recombination by using the pEASY-Basic Seamless Cloning and Assembly Kit (Transgen, China) according to the manufacturer’s instructions. Three fragments were first amplified from L1-RPS. Fragment 1 contains the sequence from the beginning of 5’-UTR to the end of ORF2, while fragments 2 and 3 are the 3′-UTR fragments flanking the antisense EGFP expression cassette in the L1-RPS fragment. Another DNA fragment (fragment 4, which encodes the TAP tag) was synthesized by Generay Biotech Co., Ltd. (Shanghai, China), according to the sequence from the pMSCV-TAP plasmid [10]. All fragments were then inserted into pcDNA6/myc-His B vector (Invitrogen, CA) through DNA recombination in the order of 1–4–2-3 to generate L1–2TAP, containing a complete L1_RP_-based LINE-1 sequence that expresses a TAP-tagged ORF2p.

Similar DNA recombination method was also used to generate the CMV-driving LINE-1 reporter plasmid ZY101. A fragment of L1-RPS was amplified through standard PCR, which contains the sequence from the beginning of ORF1to the end of 3′-UTR, including the inserted antisense EGFP expressing cassette. The amplified fragment was then inserted into pcDNA3.1(−) vector (Invitrogen) with the help of the pEASY-Basic Seamless Cloning and Assembly Kit.

All transfections were performed with Lipofectamine 3000 (Sigma-Aldrich, USA) and Opti-MEM (Thermo Fisher Scientific, USA) according to the manufacturer’s protocol.

### Antibodies

The following antibodies were used to detect protein expression in this study: anti-tubulin (TransGen, China), anti-HA (Thermo Fisher Scientific, USA), anti-myc (Sigma-Aldrich, USA), anti-TAP (Thermo Fisher Scientific, USA), anti-ORF1p (Millipore, USA), anti-IFNb1 (Abcam, UK) and anti-Histone (GenScript, China). All antibodies were used according to the manufacturer protocols.

### LINE-1 retrotransposition assay

The LINE-1 retrotransposition assay has been previously described [33, 51]. In brief, the LINE-1 plasmid (i.e., L1-RPS or ZY101) was transfected into HEK293T cells at 2 μg per well in 12-well plates together with VR1012 or one of the test plasmids. The cells were selected by the addition of puromycin (final concentration, 5 μg/ml) at 48 h post-transfection. GFP-positive cells were examined 48 h later by flow cytometry using a FACSCalibur cytometer. Gating exclusions were based on background fluorescence of the retrotransposition-incompetent JM111. At least 20,000 single-cell events per sample were gated and analysed using FlowJo (version x.0.7).

### Luciferase assay

The dual-luciferase reporter system from Promega was used to detect potential effects on promoter activity. However, only firefly luciferase was used and checked for activity, to avoid any possible impact of MDA5 on other promoters, which might compromise the authenticity of final results. In brief, one of above luciferase-expressing vectors (5UTR-Luc, IFNB-Luc, MCSFR-Luc, or RSV-Luc) was co-transfected with one of the tested plasmids or the control plasmid (VR1012) into HEK293T cells. At 48 h post-transfection, the cells were lysed with TransDetect Double-Luciferase Reporter Assay Kit (Transgen, China) and tested with a Fluoroskan Ascent™ FL Microplate Fluorometer and Luminometer (Thermo Fisher Scientific, USA) according to manufacturer’s protocol for luciferase detection. The empty vector pGL3-basic was used as negative control for luciferase activity, and the results were used to remove background noise (and are not shown).

### Western blotting

Interested cells were lysed in 1× loading buffer (80 mM Tris, pH 6.8, with 2.0% sodium dodecyl sulfate [SDS], 10% glycerol, 100 mM dithiothreitol, and 0.2% bromophenol blue). The samples were boiled for 30 min, and proteins were separated by SDS-polyacrylamide gel electrophoresis (PAGE). Proteins were transferred onto nitrocellulose membranes (GE Whatman, UK) and probed with various primary antibodies against the indicated proteins (see the figure legends). Secondary antibodies were alkaline phosphatase-conjugated anti-rabbit (Jackson, USA) and anti-mouse (Jackson, USA), and staining was carried out with 5-bromo-4-chloro-3-indolylphosphate (BCIP) (Roche, Switzerland) and nitroblue tetrazolium (NBT) (Sigma-Aldrich, USA). For each lane of protein gels, 30 μg total protein was loaded for the detection of exogenous proteins and loading controls, while 50 μg was loaded for the detection of endogenous proteins.

### RNA isolation, reverse transcription, and PCR amplification

Extraction of total RNA from transfected cell samples was performed using FastPure Cell/Tissue Total RNA Isolation Kit (Vazyme, China) according to the manufacturer’s instructions. The cDNA was generated using MonSciptTM RT III All-in-One Mix (with DNase) (Monad, China), according to the provided instructions. PCRs were performed using 2*Phanta Max Master Mix (Vazyme, China) according to the manufacturer’s instructions. The reactions were performed under the following conditions as suggested by the manufacturer: 95 °C for 30 s, followed by 28 cycles of 95 °C for 5 s, 56 °C for 30 s, and 72 °C for 45 s, then followed by 72 °C for 10 min.The primers used for PCR were as follows: ACTB, forward (5′-ACCGAGCGCGGCTACAG-3′) and reverse (5′-CTTAATGTCACGCACGATTTCC-3′); L1–3, forward (5′-CAAACACCGCATATTCTCACTCA-3′, corresponding to position 5777–5799 in L1.3 sequence [38]) and EFGP-2F (5′-ACTACCTGAGCACCCAGTCC-3′). The expected length of the amplified L1-RPS fragment is ~ 600 bp.

### Quantitative real-time reverse transcription PCR (qRT-PCR)

At 48 h post-transfection, the cells were subjected to quantitative real-time PCR (qRT-PCR) to determine the changes in endogenous mRNA levels. RNA from the samples of interest was first extracted with a FastPure Cell/Tissue Total RNA Isolation Kit (Vazyme, China) and then subjected to reverse transcription with MonSciptTM RT III All-in-One Mix (with DNase) (Monad, China). The qRT-PCR was performed with TransStart Top Green qPCR SuperMix (Transgen, China) and specific primers. The reaction was performed under the following conditions as suggested by the manufacturer: 94 °C for 30 s, followed by 40 cycles at 94 °C for 10 s and 60 °C for 30 s, followed by a dissociation protocol. Single peaks in the melting curve analysis indicated specific amplicons. The primers used for qRT-PCR were as follows: ACTB, forward (5′-ACCGAGCGCGGCTACAG-3′) and reverse (5′-CTTAATGTCACGCACGATTTCC-3′); IFNB, forward (5′-CACGACAGCTCTTTCCATGA-3′) and reverse (5′-AGCCAGTGCTCGATGAATCT-3′); OAS2, forward (5′-TTCTGCCTGCACCACTCTTCAACGA-3′) and reverse (5′- GCCAGTCTTCAGAGCTGTGCCTTTG-3′); OAS3, forward (5′-CCGAACTGTCCTGGGCCTGATCC-3′) and reverse (5′-CCCATTCCCCAGGTCCCATGTGG-3′); MX2, forward (5′-CAGAGGCAGCGGAATCGTAA-3′) and reverse (5′-TGAAGCTCTAGCTCGGTGTTC-3′); L1–1, forward (5′-GAATGATTTTGACGAGCTGAGAGAA-3′, corresponding to position 1013–1037 in L1.3 sequence) and reverse (5′-GTCCTCCCGTAGCTCAGAGTAATT-3′, position 1056–1079); IFIH1, forward (5′-AGGAGTCAAAGCCCACCATCT-3′) and reverse (5′-GGTGACGAGACCATAACGGATAA-3′); the luciferase gene (Luc), forward (5′-GAGATACGCCCTGGTTCCTG-3′) and reverse (5′-TGCATACGACGATTCTGTGATT-3′). The endogenous mRNA levels of ACTB were used as the loading control and are not shown unless otherwise indicated.

### HIV-1 production and infection

NL4–3 Δenv EGFP (a generous gift of Dr. R. Siliciano) [50] was co-transfected together with pHEF-VSVG (from Dr. L.-J. Chang through the AIDS Research and Reference Reagent Program, Division of AIDS, NIAID, NIH) into HEK293T cells. The medium was changed at 24 h post-transfection, and the supernatant was collected after an additional 24 h. For viral infection, equal volumes of viruses were used to infect THP-1 cells seeded onto a 12-well plate in the presence of DEAE (Sigma-Aldrich, USA) at a final concentration of 20 μg/ml. The cells were collected and analysed for GFP expression using a FACSCalibur (BD Biosciences, USA); 20,000 single-cell events per sample were gated and analysed using FlowJo (version x.0.7).

### Knockdown of endogenous MDA5 expression

To reduce endogenous *IFIH1* RNA levels (which encodes MDA5), cells were transfected with *IFIH1*-specific siRNA at a total final concentration of 100 nM using Lipofectamine RNAiMax (Invitrogen). The siRNAs used in this study were designed and synthesized by RiboBio Co., Ltd. (Guangzhou, China). The target sites of genes of these siRNAs are siIFIH1–1, 5′-GCCTGGAAAAGTTATAGTT-3′; siIFIH1–2, 5′-GTATCGTGTTATTGGATTA-3′. A non-targeting siRNA (RiboBio) was used as the negative control.

### Co-immunoprecipitation (co-IP)

The co-IP experiments were performed as previously reported [9]. HEK293T cells were co-transfected with 5UTR-Luc and the vector expressing MDA5-myc or YY1-myc. The cells were then harvested at 48 h post-transfection, washed with 1× phosphate-buffered saline and suspended in lysis buffer (50 mM Tris–HCl [pH 7.5], 150 mM NaCl and 0.5% NP-40, supplemented with Roche protease inhibitor cocktail). Samples were sonicated at 20% power for 60 s with a 3 s break every 3 s and then centrifuged to obtain a clear supernatant. Input samples were incubated with a myc antibody and protein A/G(MCE, USA) for 3 h, then washed several times with wash buffer (20 mM Tris-HCl [pH 7.5], 100 mM NaCl, 0.1 mM ethylenediaminetetraacetic acid (EDTA) and 0.05% Tween 20). The samples were then eluted with 100 mM glycine-HCl (pH 2.5) and divided into two parts. One part was used for Western blotting experiments to determine relative levels of isolated MDA5-myc and YY1-myc, while the other part was subjected to DNA isolation using QIAamp DNA Mini kit (QIAGEN) based on the manufacturer’s instructions. Finally, isolated DNA was subjected to qRT-PCR for 5UTR-Luc detection, with primers specifically targeting the luciferase gene.

### Quantification and statistical analysis

Flow cytometry data are presented as the means ± SD of three replicates within one experiment and are representative of at least three independent experimental repeats. Data were analysed using unpaired, two-tailed, Student’s *t*-tests. Differences in means were considered statistically significant at *p* < 0.05. Analyses were performed using Microsoft Excel software (Redmond, USA).

## Supplementary Information

**Additional file 1: Fig. S1.** Original flow cytometry images for Fig. 1. A. Flow cytometry images for Fig. 1C. B. Flow cytometry images for Fig. 1G. **Fig. S2.** Exogenous MDA5-myc does not affect the activity of the *MCSFR* promoter or the RSV promoter. A and C. Schematic representations of the MCSFR-Luc and the RSV-Luc cassettes, both of which were generated with the backbone vector pGL3-Basic. B and D. Luciferase activity data indicating the potency of MDA5-myc in regulating the activity of the *MCSFR* promoter and the RSV promoter. HEK293T cells seeded in a 24-well plate were co-transfected with 200 ng of MCSFR-Luc or RSV-Luc and control vector VR1012 (225 ng) or MDA5-myc-expressing vector (25, 75, or 225 ng). Luciferase activity was tested at 48 h post-transfection. The western blotting results show the MDA5-myc protein levels in the transfected cells. **Fig. S3.** Representative western blotting results for Fig. 4. **Fig. S4.** MDA5-myc moderately suppresses the retrotransposition of ZY101. A. Schematic representation of the LINE-1 expression cassette in ZY101, which basically replaces LINE-1 5′-UTR with the CMV promoter comparing to that in L1-RPS. In addition, the backbone vector was changed into pcDNA3.1(−), which is not shown. B. Flow cytometry results showing the efficacy of exogenous MDA5-myc in ZY101 suppression. HEK293T cells seeded on a 24-well plate were co-transfected with 1 μg of ZY101 and control vector VR1012 (225 ng) or MDA5-myc-expressing vectors (25, 75, or 225 ng), and were collected at 96 h post-transfection to detect EGFP-positive cells through flow cytometry. The western blotting results indicate the MDA5-myc protein levels in the transfected cells. **Fig. S5.** 2CARD-mediated IFN promotion barely contributes to its efficacy in LINE-1 suppression. A. Luciferase activity data indicating the potency of wild-type MDA5-myc or its mutants in IFNβ elevation. HEK293T cells seeded in a 24-well plate were co-transfected with 100 ng of IFNB-Luc and 45 ng of control vector VR1012 or one of MDA5-myc-expressing vectors (5, 15, or 45 ng). Luciferase activity was tested at 48 h post-transfection. The western blotting results above indicate the MDA5 protein levels in transfected cells. B. Flow cytometry results showing the efficacy of MDA5-myc mutants in L1-RPS suppression. HEK293T cells seeded on a 24-well plate were co-transfected with 1 μg of L1-RPS and control vector VR1012 (225 ng) or one of MDA5-myc-expressing vectors (25, 75, or 225 ng), and were collected at 96 h post-transfection to detect EGFP-positive cells through flow cytometry. The western blotting results indicate the MDA5-myc protein levels in the transfected cells.

## Data Availability

The datasets generated and/or analysed during the current study are available in the ZENODO repository, 10.5281/zenodo.5842735.
