# Supplementary material for: RNA sensor MDA5 suppresses LINE-1 retrotransposition by regulating the promoter activity of LINE-1 5′-UTR
Source: Mob DNA. 2022 Apr 12;13:10. doi: 10.1186/s13100-022-00268-0 (PMC9003951; doi:10.1186/s13100-022-00268-0)
Supplement: Supplementary file 1 — Additional file 1: Fig. S1. Original flow cytometry images for Fig. 1. A. Flow cytometry images for Fig. 1C. B. Flow cytometry images for Fig. 1G. Fig. S2. Exogenous MDA5-myc does not affect the activity of the MCSFR promoter or the RSV promoter. A and C. Schematic representations of the MCSFR-Luc and the RSV-Luc cassettes, both of which were generated with the backbone vector pGL3-Basic. B and D. Luciferase activity data indicating the potency of MDA5-myc in regulating the activity of the MCSFR promoter and the RSV promoter. HEK293T cells seeded in a 24-well plate were co-transfected with 200 ng of MCSFR-Luc or RSV-Luc and control vector VR1012 (225 ng) or MDA5-myc-expressing vector (25, 75, or 225 ng). Luciferase activity was tested at 48 h post-transfection. The western blotting results show the MDA5-myc protein levels in the transfected cells. Fig. S3. Representative western blotting results for Fig. 4. Fig. S4. MDA5-myc moderately suppresses the retrotransposition of ZY101. A. Schematic representation of the LINE-1 expression cassette in ZY101, which basically replaces LINE-1 5′-UTR with the CMV promoter comparing to that in L1-RPS. In addition, the backbone vector was changed into pcDNA3.1(−), which is not shown. B. Flow cytometry results showing the efficacy of exogenous MDA5-myc in ZY101 suppression. HEK293T cells seeded on a 24-well plate were co-transfected with 1 μg of ZY101 and control vector VR1012 (225 ng) or MDA5-myc-expressing vectors (25, 75, or 225 ng), and were collected at 96 h post-transfection to detect EGFP-positive cells through flow cytometry. The western blotting results indicate the MDA5-myc protein levels in the transfected cells. Fig. S5. 2CARD-mediated IFN promotion barely contributes to its efficacy in LINE-1 suppression. A. Luciferase activity data indicating the potency of wild-type MDA5-myc or its mutants in IFNβ elevation. HEK293T cells seeded in a 24-well plate were co-transfected with 100 ng of IFNB-Luc and 45 ng of control vecto [file 13100_2022_268_MOESM1_ESM.docx]

Supplementary Figure Legends


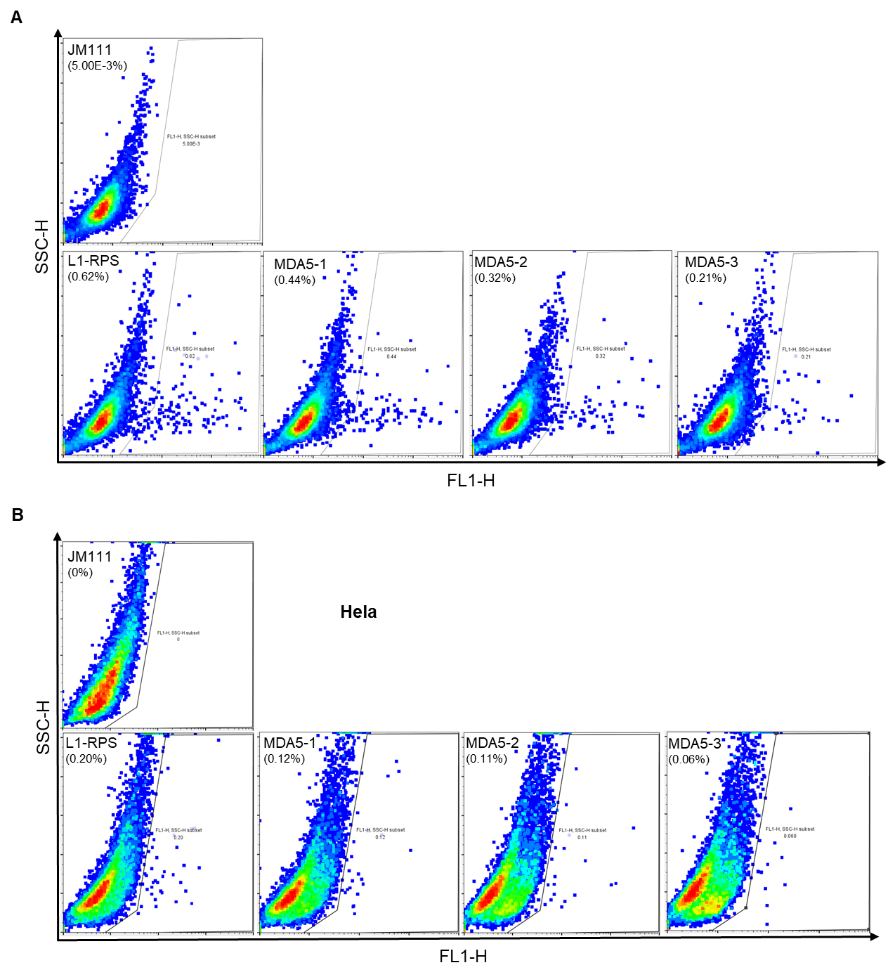


Fig.S1. Original flow cytometry images for Fig. 1. A. Flow cytometry images for Fig.1C. B. Flow cytometry images for Fig.1G.


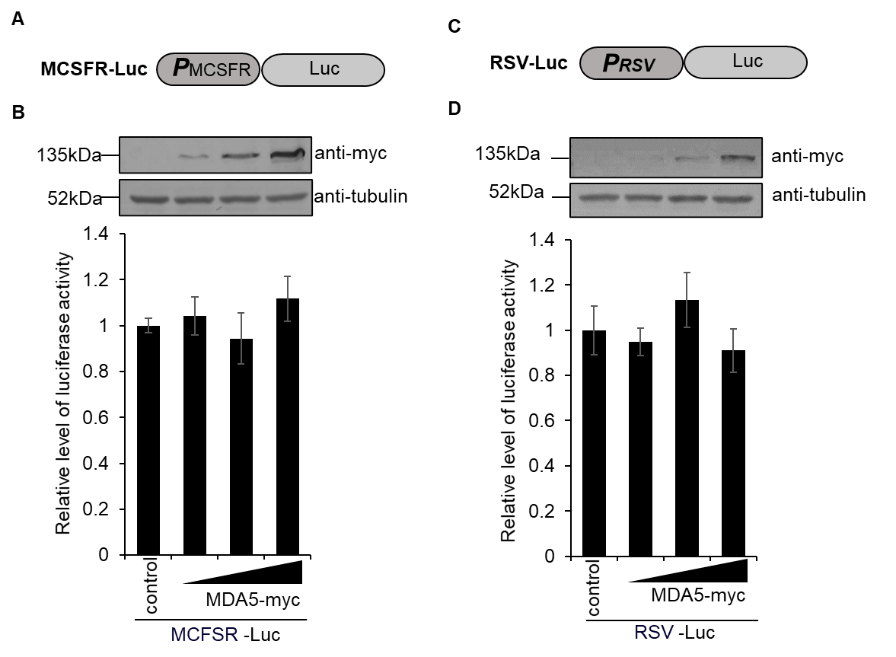


Fig.S2. Exogenous MDA5-myc does not affect the activity of the *MCSFR* promoter or the RSV promoter. A and C. Schematic representations of the MCSFR-Luc and the RSV-Luc cassettes, both of which were generated with the backbone vector pGL3-Basic. B and D. Luciferase activity data indicating the potency of MDA5-myc in regulating the activity of the *MCSFR* promoter and the RSV promoter. HEK293T cells seeded in a 24-well plate were co-transfected with 200 ng of MCSFR-Luc or RSV-Luc and control vector VR1012 (225ng) or MDA5-myc-expressing vector (25, 75, or 225 ng). Luciferase activity was tested at 48 h post-transfection. The western blotting results show the MDA5-myc protein levels in the transfected cells.


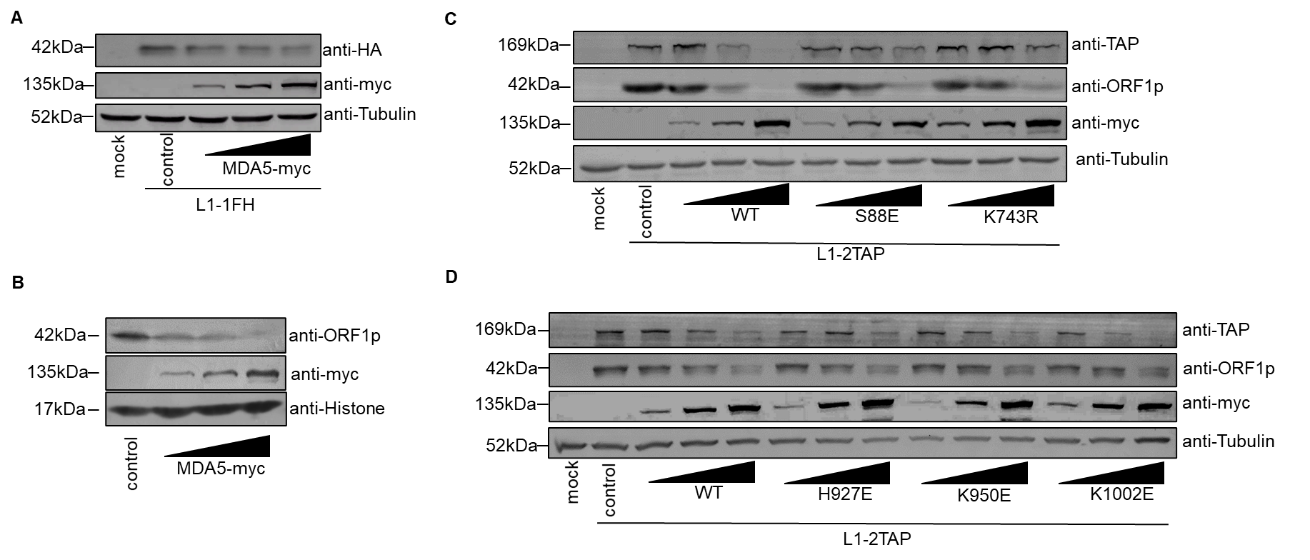


Fig.S3. Representative western blotting results for Figure 4.


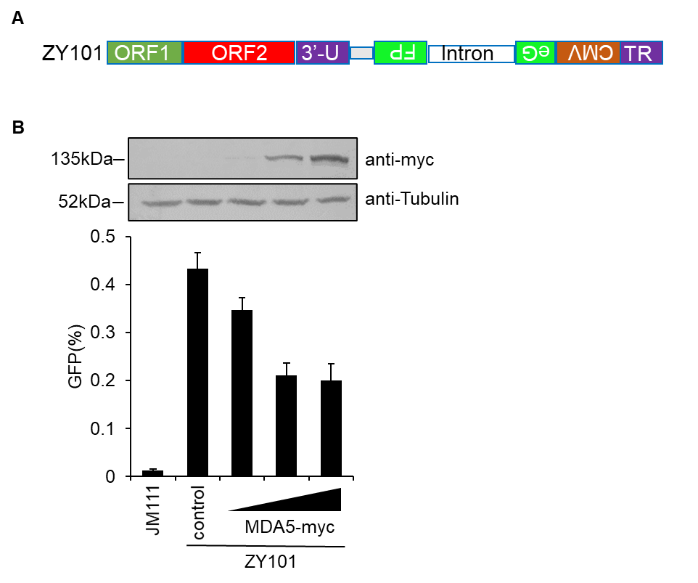


Fig.S4. MDA5-myc moderately suppresses the retrotransposition of ZY101. A. Schematic representation of the LINE-1 expression cassette in ZY101, which basically replaces LINE-1 5’-UTR with the CMV promoter comparing to that in L1-RPS. In addition, the backbone vector was changed into pcDNA3.1(-), which is not shown. B. Flow cytometry results showing the efficacy of exogenous MDA5-myc in ZY101 suppression. HEK293T cells seeded on a 24-well plate were co-transfected with 1 μg of ZY101 and control vector VR1012 (225ng) or MDA5-myc-expressing vectors (25, 75, or 225 ng), and were collected at 96 h post-transfection to detect EGFP-positive cells through flow cytometry. The western blotting results indicate the MDA5-myc protein levels in the transfected cells.


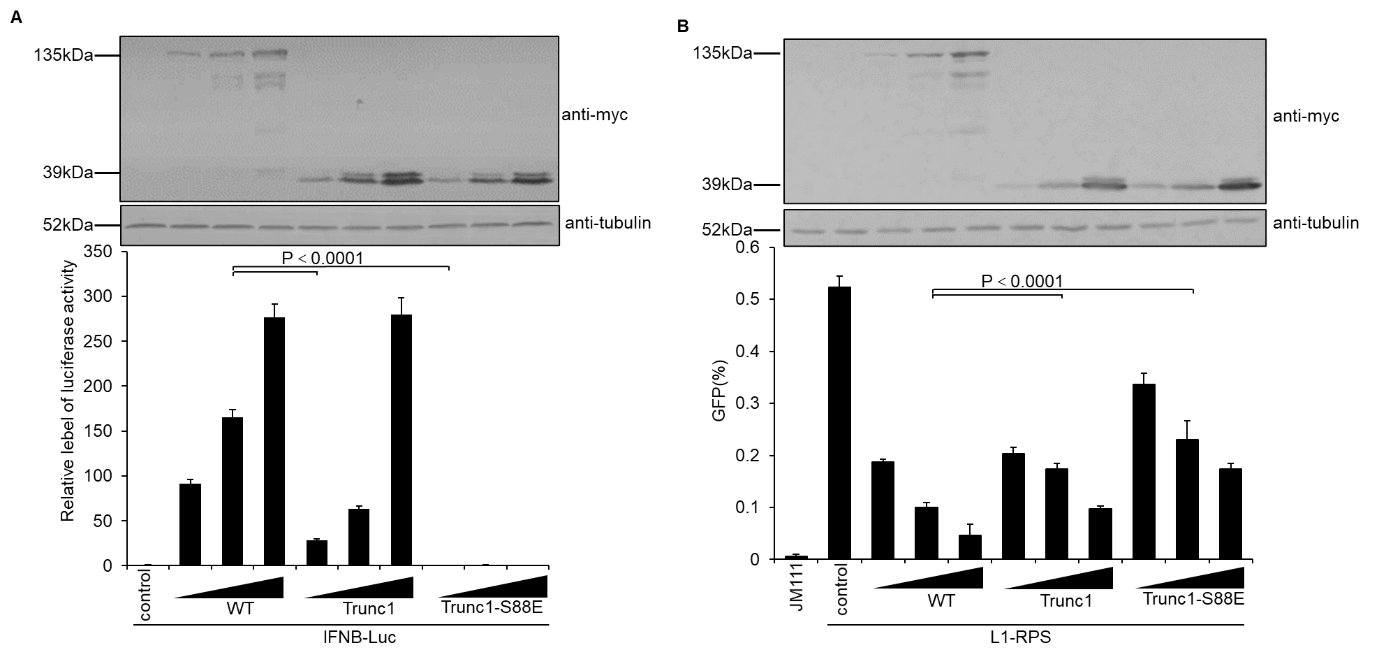


Fig.S5. 2CARD-mediated IFN promotion barely contributes to its efficacy in LINE-1 suppression. A. Luciferase activity data indicating the potency of wild-type MDA5-myc or its mutants in IFNβ elevation. HEK293T cells seeded in a 24-well plate were co-transfected with 100 ng of IFNB-Luc and 45 ng of control vector VR1012 or one of MDA5-myc-expressing vectors (5, 15, or 45 ng). Luciferase activity was tested at 48 h post-transfection. The western blotting results above indicate the MDA5 protein levels in transfected cells. B. Flow cytometry results showing the efficacy of MDA5-myc mutants in L1-RPS suppression. HEK293T cells seeded on a 24-well plate were co-transfected with 1 μg of L1-RPS and control vector VR1012 (225ng) or one of MDA5-myc-expressing vectors (25, 75, or 225 ng), and were collected at 96 h post-transfection to detect EGFP-positive cells through flow cytometry. The western blotting results indicate the MDA5-myc protein levels in the transfected cells.
